# Automated segmentation of long and short axis DENSE cardiovascular magnetic resonance for myocardial strain analysis using spatio-temporal convolutional neural networks

**DOI:** 10.1186/s12968-023-00927-y

**Published:** 2023-03-30

**Authors:** Hugo Barbaroux, Karl P. Kunze, Radhouene Neji, Muhummad Sohaib Nazir, Dudley J. Pennell, Sonia Nielles-Vallespin, Andrew D. Scott, Alistair A. Young

**Affiliations:** 1 School of Biomedical Engineering and Imaging Sciences, King’s College London, London, UK; 2 Cardiovascular Magnetic Resonance Unit, The Royal Brompton Hospital (Guy’s and St Thomas’ NHS Foundation Trust), London, UK; 3 National Heart and Lung Institute, Imperial College London, London, UK; 4 MR Research Collaborations, Siemens Healthcare Limited, Camberley, UK

**Keywords:** Cardiac, MRI, Strain, DENSE, Deep learning, Spatio-temporal, Segmentation

## Abstract

**Background:**

Cine Displacement Encoding with Stimulated Echoes (DENSE) facilitates the quantification of myocardial deformation, by encoding tissue displacements in the cardiovascular magnetic resonance (CMR) image phase, from which myocardial strain can be estimated with high accuracy and reproducibility. Current methods for analyzing DENSE images still heavily rely on user input, making this process time-consuming and subject to inter-observer variability. The present study sought to develop a spatio-temporal deep learning model for segmentation of the left-ventricular (LV) myocardium, as spatial networks often fail due to contrast-related properties of DENSE images.

**Methods:**

2D + time nnU-Net-based models have been trained to segment the LV myocardium from DENSE magnitude data in short- and long-axis images. A dataset of 360 short-axis and 124 long-axis slices was used to train the networks, from a combination of healthy subjects and patients with various conditions (hypertrophic and dilated cardiomyopathy, myocardial infarction, myocarditis). Segmentation performance was evaluated using ground-truth manual labels, and a strain analysis using conventional methods was performed to assess strain agreement with manual segmentation. Additional validation was performed using an externally acquired dataset to compare the inter- and intra-scanner reproducibility with respect to conventional methods.

**Results:**

Spatio-temporal models gave consistent segmentation performance throughout the cine sequence, while 2D architectures often failed to segment end-diastolic frames due to the limited blood-to-myocardium contrast. Our models achieved a DICE score of 0.83 ± 0.05 and a Hausdorff distance of 4.0 ± 1.1 mm for short-axis segmentation, and 0.82 ± 0.03 and 7.9 ± 3.9 mm respectively for long-axis segmentations. Strain measurements obtained from automatically estimated myocardial contours showed good to excellent agreement with manual pipelines, and remained within the limits of inter-user variability estimated in previous studies.

**Conclusion:**

Spatio-temporal deep learning shows increased robustness for the segmentation of cine DENSE images. It provides excellent agreement with manual segmentation for strain extraction. Deep learning will facilitate the analysis of DENSE data, bringing it one step closer to clinical routine.

**Supplementary Information:**

The online version contains supplementary material available at 10.1186/s12968-023-00927-y.

## Background

Myocardial deformation holds important diagnosis and prognosis value for the assessment of heart conditions [45], which remains one of the largest causes of death worldwide [5]. In particular, myocardial strain provides significant added value compared to the left ventricular (LV) ejection fraction (LVEF) [41, 52]. LVEF is a ubiquitous biomarker for the assessment of cardiac conditions, but is limiting when we need to understand underlying mechanisms, and cannot provide regional information [2, 30, 57]. A normal LVEF can mask cardiac dysfunction and lead to missed or delayed detection of disease. Myocardial strain has been shown to be particularly useful for the diagnosis of congenital heart diseases [9] and prediction of cardiac function after repair [24, 53], while severe regional strain impairment correlates with myocardial infarction (MI; [10, 34, 35]. Strain assessment also has added value for diagnosis [15, 36, 46] and prognosis [38] of cardiomyopathies, and is being recommended for cardiotoxicity analysis after cancer treatment [17, 40, 51, 55]. More recently, the assessment of myocardial strain is recommended in international guidelines for the care of cardio-oncology patients [32].

While myocardial strain can be estimated from a range of modalities and acquisition protocols, cardiovascular magnetic resonance (CMR), and in particular cine displacement encoding with stimulated echoes (DENSE) imaging, is a promising technique for strain assessment [1, 29, 64], and has been shown highly reproducible [60]. The adoption of echocardiography-based methods, like tissue Doppler imaging and speckle tracking, can be limited due to the need for highly trained operators [8]. Other CMR techniques, like tissue tagging and phase-velocity encoding, have also shown some limitations in their ability to provide accurate strain estimation with high spatial resolution [1, 20]. Feature tracking CMR using standard cines does not reliably quantify *regional* strain [56].

The application of DENSE to clinical pipelines is limited by the lack of automation for the extraction of myocardial strain. Current tools like DENSEanalysis [12, 47] require significant human interaction in contouring the LV epicardial and endocardial borders. In DENSE images, displacement information is encoded in the image phase, and while the most advanced algorithms are able to automatically unwrap the phase and extract Lagrangian strain from pixel displacements, these steps generally still require human adjustments, either to select specific hyper-parameters or guide phase-unwrapping by selecting seed points in non-wrapped areas of the phase.

The use of deep learning (DL) for cardiovascular imaging, and in particular convolutional neural networks, has been significantly increasing in the past years. These models are being used extensively for CMR segmentation [22, 28, 42, 43, 61, 61]. Recently, nnU-Net was presented by Isensee et al*.* [21] which builds on U-Net, the segmentation model developed by Ronneberger et al*.* [44] in 2015. Strategies were developed in nnU-Net to improve the performance of U-Net for a highly diverse range of medical image segmentation tasks, and it has been widely adopted as a benchmark method. nnU-Net optimizes some hyper-parameters used in training U-Net models, either empirically, or on a task-by-task basis given the characteristics of a given dataset. Data augmentation is also performed with a wide range of transformations, while the inference performance is boosted by implementing test-time augmentation strategies. Additionally, there is an emergence of DL methods for automated cardiac motion analysis [4, 31, 43, 59]. However, there are few studies related to DENSE imaging for post processing. Recently, Ghadimi et al*.* [11] showed, using basic 2D U-Net models, very promising results for automatic segmentation and extraction of strain from short-axis DENSE acquisitions. However, these 2D method processed each frame independently, so temporal coherence, T1 relaxation and blood flow through the slice were not incorporated, which can make segmentation of the first and last frames difficult due to lack of contrast. Also, only short-axis analyses were performed. Kar et al*.* [27] showed how this could be achieved using 2D deep CNNs, but exhibit the same limitations.

In this study, we explore the use of DL to automate the segmentation of LV myocardial short and long-axis DENSE images. In particular, we show how temporal redundancies in cine sequences (2D + time) can be leveraged to improve segmentation performance and coherence. The nnU-Net pipeline was extended in this work under the MONAI framework [37] to create models that can easily be integrated into scanner platforms and clinical pipelines. Models are validated by comparing segmentation maps to ground-truth manual contouring, both in internal and external test datasets. Further validation is performed by automatically extracting Lagrangian displacement and strain values, and quantifying variability in relation to previous studies [3]. To this purpose, we extended the DENSEanalysis Matlab tool ([12], Mathworks, Natick, Massachusetts, USA) to be able to automatically analyze studies with minimal interaction using the automated contours as input.

## Methods

### Study population

DENSE CMR examinations were obtained according to local ethical approvals with informed consent at the Royal Brompton Hospital in London, UK as part of a number of research studies [3, 16, 50]. Between April 2014 and August 2021, 260 subjects have been scanned at 3T (MAGNETOM Skyra and MAGNETOM Vida, Siemens Healthineers, Erlangen, Germany). The cohort study includes healthy subjects and patients affected by various cardiac conditions, including acute and chronic MI, dilated cardiomyopathy (DCM), hypertrophic cardiomyopathy (HCM), myocarditis, recovered DCM patients, and sickle cell. This is the primary dataset used in this study to train our models but also from which we extracted our main independent test set to validate the results. A second dataset was acquired at King’s College London in 2020 from healthy subjects, and is used in this study to study domain adaptation and further validate the results. Subjects were scanned at 1.5T and 3T (respectively on MR-PET Biograph mMR and MAGNETOM Aera, Siemens Healthineers). All data were anonymized on the hospital systems before further analysis. Demographic data can be found on Table 1.

**Table 1:** Study cohort

	Primary cohort	Secondary cohort
Subjects	N = 260	N = 11
Exams	382	31
Age at scan (y)	48.9 ± 14.9	32.9 ± 8.0
Sex (F/M/O)	69/190/1	4/7/0
Weight (kg)	81.5 ± 18.4	73.4 ± 15.8
Height (cm)	174 ± 11	173 ± 9
Healthy	57	11
Acute MI	40	0
DCM	59	0
HCM	13	0
Myocarditis	36	0
Recovered DCM	53	0
Sickle cell	1	0
Chronic MI	1	0

*F* Female, *M* Male, *DCM* Dilated Cardiomyopathy, *HCM* Hypertrophic Cardiomyopathy, *MI* Myocardial Infarction, *O* Other

### DENSE imaging protocol and image analysis

A prototype spiral cine DENSE sequence [49, 64] was used to acquire short-axis and long-axis images. Between one and three short-axis planes (basal, mid and apical) were acquired, along with up to three long-axis planes (two, three and four-chamber views). The following typical acquisition parameters were used: encoding frequency of 0.1 cycles/mm, simple three-point encoding providing 2D in-plane displacements [29, 63], through-plane dephasing of 0.08 cycles/mm, 4 spiral interleaves with a spiral readout duration of 5.5 ms providing an acquired spatial resolution of 3.4 mm in-plane, reconstructed onto a $128\times 128$ image matrix, slice thickness of 8 mm, repetition time of 15 ms, echo time of 1 ms, variable flip angle increasing to a maximum of 15° in the final frames, two spiral interleaves per frame per heartbeat acquired to give 30 ms temporal resolution, breath-hold acquisition over 14 cardiac cycles. Part of the dataset was acquired with outer volume suppression, resulting in four different fields of view: 200 mm [3], 120 mm, 224 mm and 360 mm [49].

**Fig. 1:**
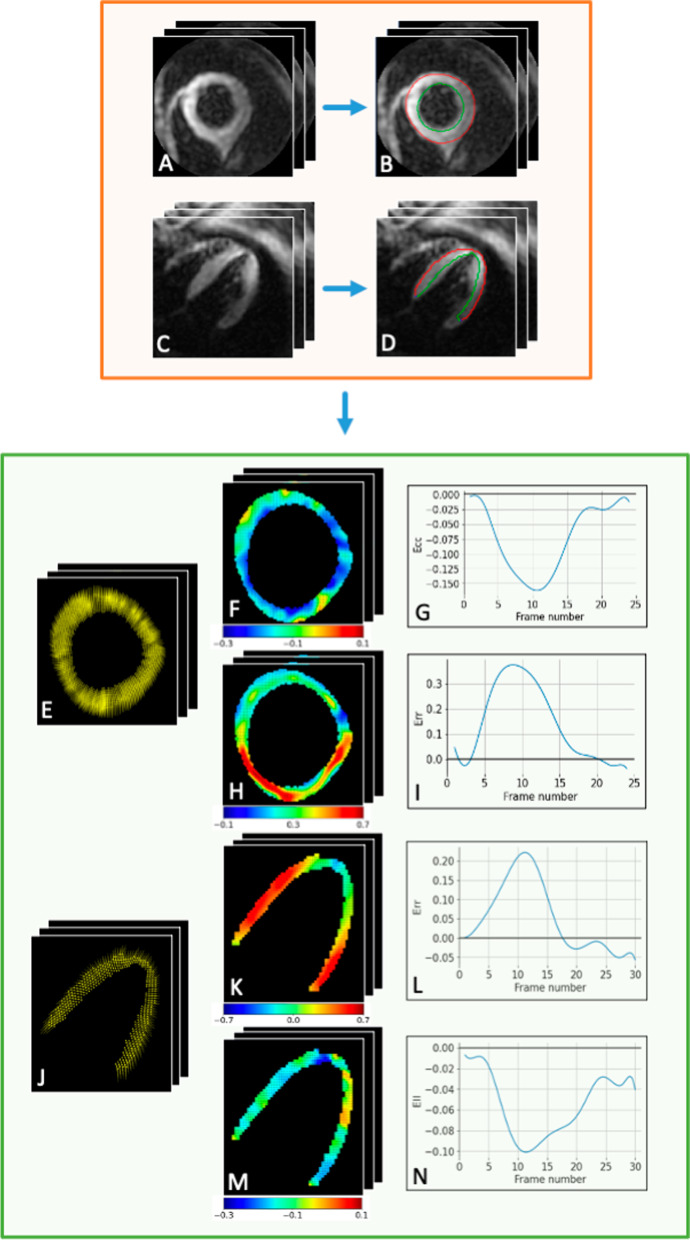
**Illustration of the processing pipeline**. The first step (orange box) is the segmentation of the left-ventricular (LV) myocardium. It is done manually with DENSEanalysis to obtain training labels, and replaced with a fully automated deep learning (DL) model in this work. Example shown here of an end-systolic short-axis magnitude frame (respectively long-axis) for a healthy subject (A, respectively C) and corresponding ground-truth manual contours (B, respectively D). Lagrangian displacement (E, respectively J) and circumferential (Ecc)/radial (Err)/longitudinal (Ell) strain components (F/H, respectively K/M) are then calculated and strain time curves produced (G/I, respectively L/N)

DENSE images were analyzed by human observers using the open-source DENSEanalysis Matlab tool (Mathworks; [12]), with different subsets of the dataset being analyzed by different observers. The LV myocardium was semi-automatically segmented using motion-guided segmentation [48], to propagate LV contours from a single frame to the rest of the DENSE cine images, followed by manual correction if needed (every case, and almost every frame in each case, required at least minimal adjustment). A phase-unwrapping algorithm (spatiotemporal quality-guided path following) was then performed on the myocardial pixels, and Langrangian displacement and strain values were calculated by DENSEanalysis, as described in [47]. Circumferential (Ecc) and radial (Err) strain components were extracted for short-axis images, while Ell and Err strain components were extracted for long-axis images. DENSE images from a healthy subject and the processing pipeline steps are illustrated on Fig. 1.

### Image and label quality control

In order to assess the quality of the images for training and the corresponding manual LV segmentation, an application was created in Python with a Graphical User Interface (GUI) to rapidly show the available images and allow rejection when quality is impaired or manual segmentation appears to insufficiently track the LV epi- and endocardium. An overview of the GUI can be found in Additional file 1: Fig. S1.

### Segmentation models and training

After quality control, the dataset was reduced to 360 short-axis and 124 long-axis slices (from 220 subjects). In each case, approximately 80% of the data (split by patient) was used for training, while 20% was left as an independent test set for performance assessment. All models were trained using a fourfold cross-validation (CV) on the training set, and the CV performance was used to optimise the networks. The CV folds were carefully engineered to maintain the dataset pathology distributions between folds. As some subjects were scanned multiple times in follow-up visits, all slices from a single subject were included in the same fold to prevent overfitting (also for the 20% performance testing dataset). A summary of the folds can be found in Tables 2 and 3 (for short-axis and long-axis datasets respectively).

**Table 2:** Fold split statistics for short-axis data. The total number of exams is different than the number of subjects as cases might have been rejected by quality-control checks and subjects might have undergone follow-up scans

	#	#	Sex			Physical characteristics			Disease types						
	exams	slices	M	F	O	Height(cm)	Weight(kg)	Age(years)	MCD	Healthy	RDCM	DCM	MI	HCM	Sickle
1	54	83	38	16	0	174±10	80.3±17.0	46.5±15.2	6	13	15	10	8	2	0
2	50	72	32	18	0	171±14	84.4±19.1	54.4±13.0	3	11	16	10	8	2	0
3	47	62	35	12	0	176±11	82.0±19.7	51.4±12.5	4	11	13	10	8	1	0
4	47	60	32	15	0	173±10	81.1±19.5	47.7±11.8	5	10	12	10	8	2	0
Test	53	83	37	15	1	174±11	80.1±18.6	48.0±13.2	5	12	15	10	8	2	1

*M* Male, *F* Female, *O* Other. *MCD* Myocarditis, *DCM* Dilated cardiomyopathy, *RDCM* Recovered DCM, *MI* Acute myocardial infarction, *HCM* Hypertrophic cardiomyopathy, *Sickle* Sickle cell

**Table 3:** Fold split statistics for long-axis data. The total number of exams is different than the number of subjects as cases might have been rejected by quality-control checks and subjects might have undergone follow-up scans

	#	# slices			Sex		Physical characteristics			Disease types					
	Exams	2ch	3ch	4ch	M	F	Height(cm)	Weight(kg)	Age(years)	MCD	Healthy	RDCM	DCM	HCM	CMI
1	23	6	1	18	17	6	177±10	78.7±20.4	48.8±13.0	3	9	9	2	0	0
2	21	5	2	19	12	9	172±13	81.3±19.8	45.2±11.6	2	8	9	2	0	0
3	20	7	2	14	13	7	174±12	84.7±18.1	51.1±13.2	3	6	9	2	0	0
4	21	9	2	14	15	6	170±17	78.9±19.7	46.9±14.6	3	9	7	2	0	0
Test	21	9	2	14	15	6	174±10	83.0±21.7	48.0±16.1	2	7	9	1	1	1

*2ch* Two-chamber, *3ch* Three-chamber, *4ch* Four-chamber. *M* Male, *F* Female. *MCD* Myocarditis, *DCM* Dilated cardiomyopathy, *RDCM* Recovered DCM, *MI* Acute myocardial infarction, *HCM* Hypertrophic cardiomyopathy, *CMI* Chronic myocardial infarction

Automated segmentation of the LV myocardium from DENSE magnitude images was performed using nnU-Net with the manual segmentations from DENSEanalysis used as the ground truth. For this purpose, manual LV floating-point contours were converted into binary masks. Two nnU-Net architectures have been trained for this study: one with a 2D network, where DENSE frames are individually processed, and the other with a 2D + time network, where the cine sequences are stacked into a 3D volume and processed by a 3D network.

The nnU-Net pipeline has many automated features around its core model training. However, it offers limited options for export and portability, can be difficult to customize, and is challenging to deploy on scanners where Python environments are not available. We re-implemented and simplified nnU-Net using the MONAI library [37], based on the DynU-Net implementation. This allowed us to easily extract the trained networks for use in inference on other platforms. In this study, we also validated the performance of our MONAI pipeline implementation with regard to the original nnU-Net pipeline. Architecture and implementation details can be found in Additional file 1: Fig. S3.

### Strain analysis

After the DENSE images in the test sets were automatically segmented using the trained DL networks, the segmentation masks were converted into contours and loaded on DENSEanalysis. The ground-truth manual contours obtained with DENSEanalysis are floating-point contours, which cannot be obtained from pixel masks as provided by the network. As a result these manual contours were transformed into pixel masks and converted to pixel contours to imitate the post-processing step applied to the automated segmentations. This step removes any systematic error which may be introduced by comparing the strain results obtained from floating-point contours to those from pixel masks, allowing an analysis of the underlying differences between manually vs. automatically segmenting the LV myocardium. An analysis of the impact of using pixel contours instead of floating-point ones can be found in Additional file 1: Fig. S2.

Next, Lagrangian strain and displacement were calculated from the phase images for both ground-truth and DL-based automatic segmentations. For that purpose, the DENSEanalysis tool was modified to allow semi-automatic processing, allowing analysis of the cases in the test sets in under 30 s per case. The modifications were as follows: loading the cases in DENSEanalysis and exporting the data after analysis was made automatic; the manual selection of seed points for phase unwrapping was accelerated by initially positioning points at standardized locations in the cine DENSE dataset; the temporal fitting model (10th-order polynomial) and spatial smoothing (0.9) parameters were pre-set; most GUI interactions were removed. The user only needs to adjust the seed points if need be, which happened in a minority of cases (5%), and adjust the right-ventricular (RV) insertion points for short-axis slices.

Finally, strain results obtained from ground-truth manual contours and automated segmentation were compared. To evaluate these results, Bland–Altman analyses were performed, and the reproducibility measures coefficient of variation (CoV) and intra-class correlation coefficient (ICC) were calculated between the peak strain results obtained from the automated and manual ground-truth contours. As defined in the recent multi-center DENSE reproducibility study [3], agreement between strains from the manual and automated contours is considered excellent for CoV $\le 10\mathrm{\%}$, good for $10\mathrm{\%}<$ CoV $\le 20\mathrm{\%}$, fair for $20\mathrm{\%}<$ CoV $\le 40\mathrm{\%}$, and poor for CoV $>40\mathrm{\%}$. Similarly, agreement is considered excellent for ICC $\ge 0.74$, good for $0.6<$ ICC $\le 0.74$, fair for $0.4<$ ICC $\le 0.6$, and poor for ICC $<0.4$. Ecc and Err were calculated for short-axis images, while Ell and Err were for long-axis ones.

### Generalisation and reproducibility analysis

To assess the performance of the automated segmentation and the potential of automated segmentation as a method to reduce one contributing factor to inter-scan variability of DENSE-derived strain measures, the pipeline described above was tested on a second test set acquired at a second center (see Study Population section). Subjects were scanned on two different scanners, allowing for an assessment of the inter-scanner variability analysis. On each scanner, subjects were scanned twice, after repositioning inside the scanner, which allowed for analyzing inter-scan variability. 45 short-axis slices were manually segmented using DENSEanalysis, and were used to assess the performance of our 2D + time DynU-Net. In this work, we also compare the variability in measuring strain when the data was manually segmented or automatically estimated with DL.

### Statistics

Model performance was assessed using Dice score and Hausdorff distance (HD) between the automated segmentation and the manual ground-truth. Dice and HD are generally well-suited metrics for assessing semantic segmentation. More importantly, HD is independent of commonly used training loss measures. As a boundary-based metric, it provides a distance-based penalization of inferred structures (particularly relevant for long-axis shapes). It is also highly sensitive to outliers, as it is a measure of maximum error [33]. Two-tailed paired Student t-tests were performed with a significance level of p = 0.01, or Wilcoxon tests when the normality assumption did not hold. Statistical analyses were performed using Python (version 3.9, Python Software Foundation, Wilmington, Delaware, USA) with Scipy library (version 1.7.1).

## Results

### 2D + time analysis

**Fig. 2:**
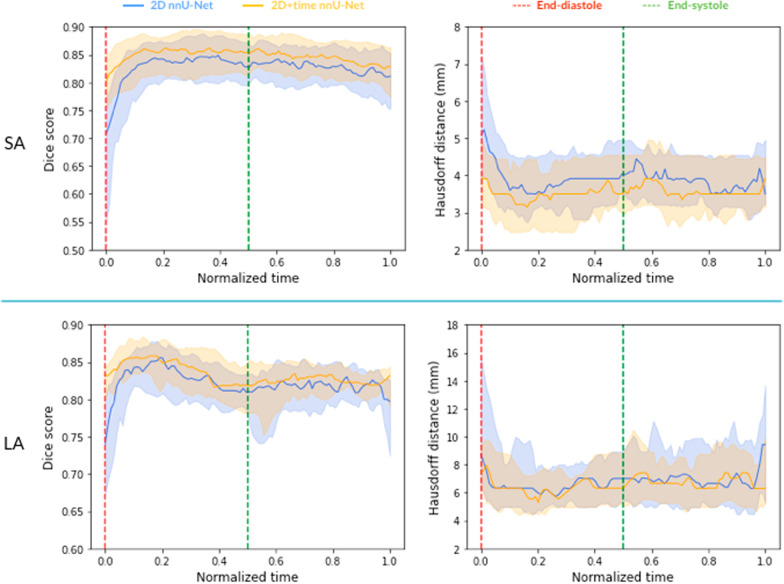
2D vs 2D + time nnU-Net performance. Performance metrics over the cardiac cycle, aggregated over an independent test set. Time was normalized to represent systole between 0 and 0.5 and diastole between 0.5 and 1. Area: interquartile range. Dark line: median

**Fig. 3:**
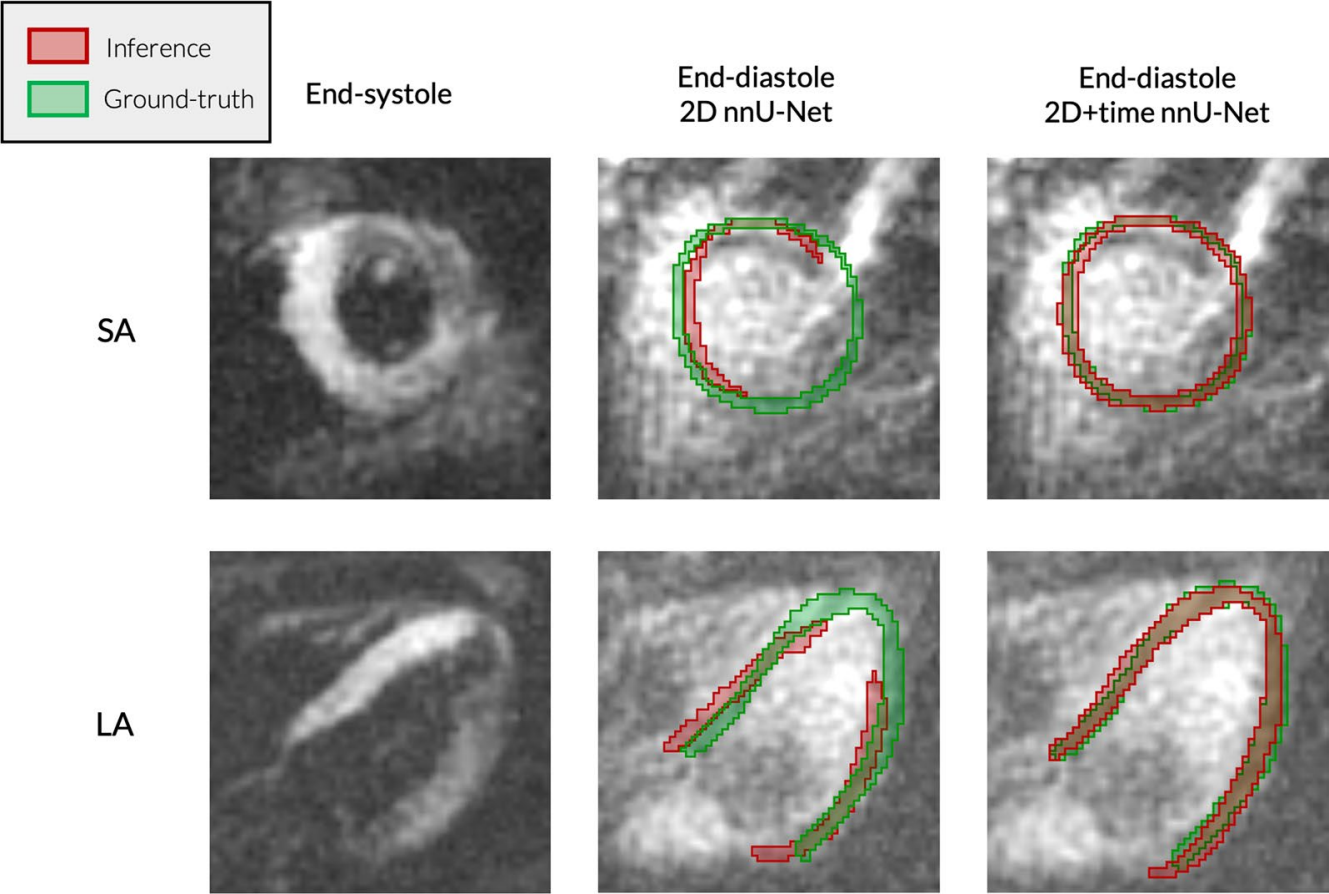
Segmentation inference examples, 2D vs 2D + time nnU-Net. Examples of end-diastolic short-axis (SAx) and long-axis (LAx) segmentation results on cases from an independent test set. For each case, 2D (middle column) and 2D + time (right column) segmentation maps are compared. The left column shows the corresponding end-systolic frame, showing better blood-to-myocardium contrast

We evaluated the performance of the two nnU-Net architectures on the first test set, frame by frame. As Fig. 2 shows, the performance of the 2D models on end-diastolic frames drops substantially with respect to the rest of the cardiac cycle, which is greatly mitigated when using the 2D + time models. The latter have a significantly improved Dice (p $<{10}^{-11}$) and Hausdorff distance (p $<{10}^{-15}$) at end-diastole. Figure 3 shows typical segmentation results for end-diastolic frames, and illustrates how segmentation can be severely impaired when using 2D models.

The short-axis 2D architecture achieved a mean Dice score of 0.82 ± 0.05 and Hausdorff distance of 4.1 ± 1.0 mm over the whole test set, while the 2D + time architecture outperformed the 2D architecture with a Dice score of 0.84 ± 0.04 (p $<{10}^{-9}$) and Hausdorff distance of 3.8 ± 1.0 mm (p $<{10}^{-7}$). Respectively, the long-axis models achieved a Dice score of 0.81 ± 0.03 and 0.82 ± 0.03 (p $<0.002$), and a Hausdorff distance of 8.2 ± 4.0 mm and 7.5 ± 3.3 mm (p $>0.01$). All the metrics are provided in Table 4.

**Table 4:** Segmentation result metrics on independent test-sets, from nnU-Net architectures (2D and 2D + time)

		DICE	HD (mm)	Precision	Sensitivity
SAx	2D	0.82 ± 0.05	4.1 ± 1.0	0.82 ± 0.08	0.84 ± 0.07
	2D + t	0.84 ± 0.04	3.8 ± 1.0	0.83 ± 0.08	0.86 ± 0.07
LAx	2D	0.81 ± 0.03	8.2 ± 4.0	0.83 ± 0.07	0.80 ± 0.07
	2D + t	0.82 ± 0.03	7.5 ± 3.3	0.83 ± 0.08	0.83 ± 0.06

*SAx* Short-axis. *LAx* Long-axis. *HD* Hausdorff distance

### Segmentation results

**Fig. 4:**
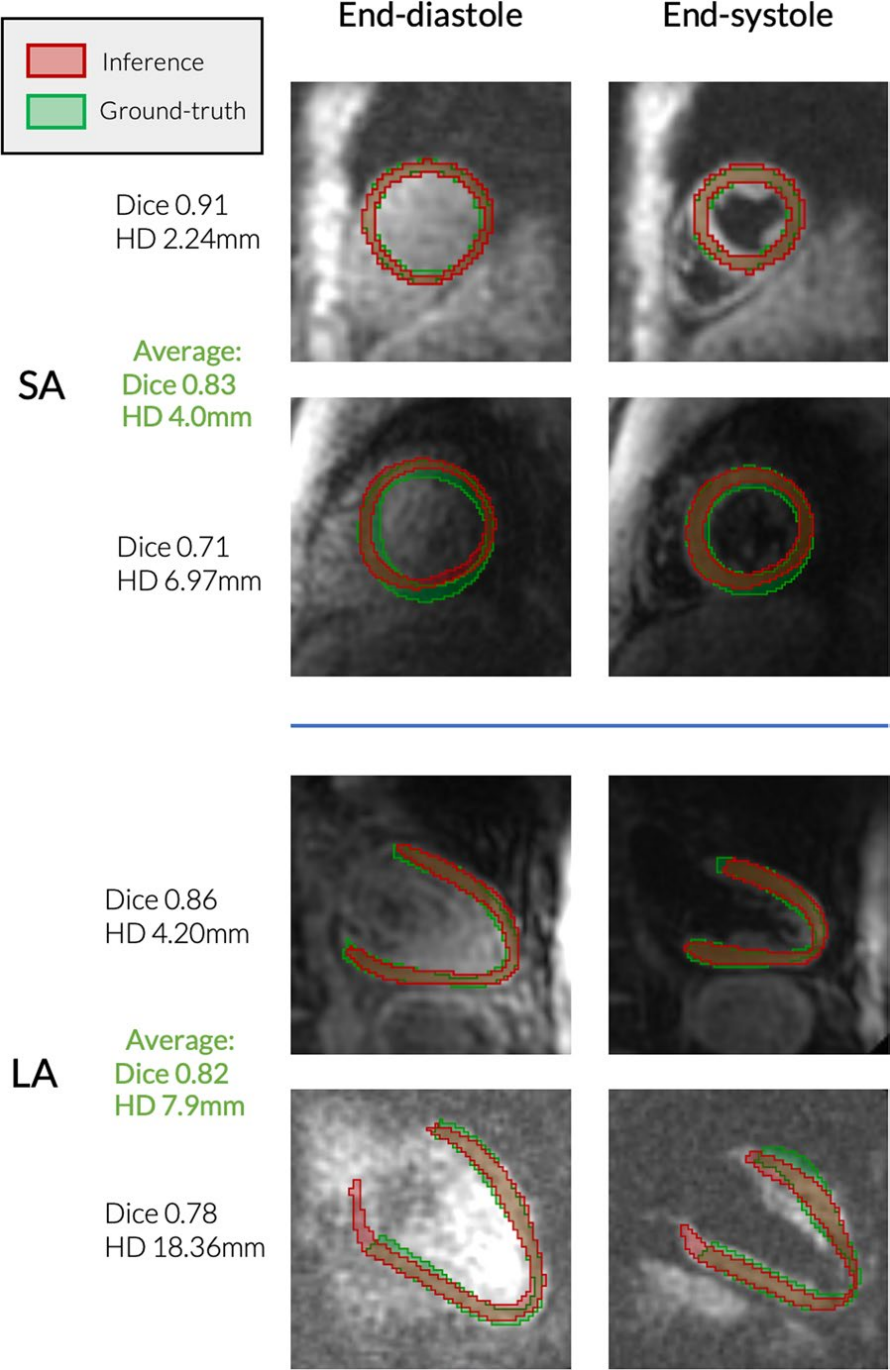
Segmentation inference examples, 2D + time DynU-Net. Examples of short-axis (SAx) and long-axis (LAx) segmentation results on cases from an independent test set. For each case, the bottom row contains inference examples of worst performance at end-diastole (left column) and end-systole (right column), while the top row contains inference examples with top performance, more typical

2D + time DynU-Net segmentation inference on an independent test-set of 83 short-axis and 25 long-axis slices achieved a DICE score of 0.83 ± 0.05 and a Hausdorff distance of 4.0 ± 1.1 mm for the short-axis and 0.82 ± 0.03 and 7.9 ± 3.9 mm respectively for the long axis. This led to a similar segmentation performance to the original nnU-Net implementation, with no statistical difference for LAx models (p $>0.01$ for Dice and Hausdorff distance on the overall test set) and a minor difference for SAx models (p $<0.003$). Additional precision and sensitivity measures can be found in Table 5. Figure 4 shows segmentation results from both LAx andSAx test sets. The training time was reduced by a factor 4 compared to the nnU-Net framework, reducing this time from 83 to 21 h for fourfold CV training on the SAx dataset, and respectively from 51.5 h to 13 h for LAx. GPU inference was achieved in under 1 s for every case, compared to up to 30 min required to define contours with manual segmentation.

**Table 5:** 2D + time segmentation result metrics on independent test-sets

		DICE	HD (mm)	Precision	Sensitivity
SAx	nnU-Net	0.84 ± 0.04	3.8 ± 1.0	0.83 ± 0.08	0.86 ± 0.07
	DynU-Net	0.83 ± 0.05	4.0 ± 1.1	0.82 ± 0.08	0.86 ± 0.07
LAx	nnU-Net	0.82 ± 0.03	7.5 ± 3.3	0.83 ± 0.08	0.83 ± 0.06
	DynU-Net	0.82 ± 0.03	7.9 ± 3.9	0.82 ± 0.07	0.82 ± 0.05

*SAx* Short-axis. *LAx* Long-axis. *HD* Hausdorff distance

### Strain analysis

**Fig. 5:**
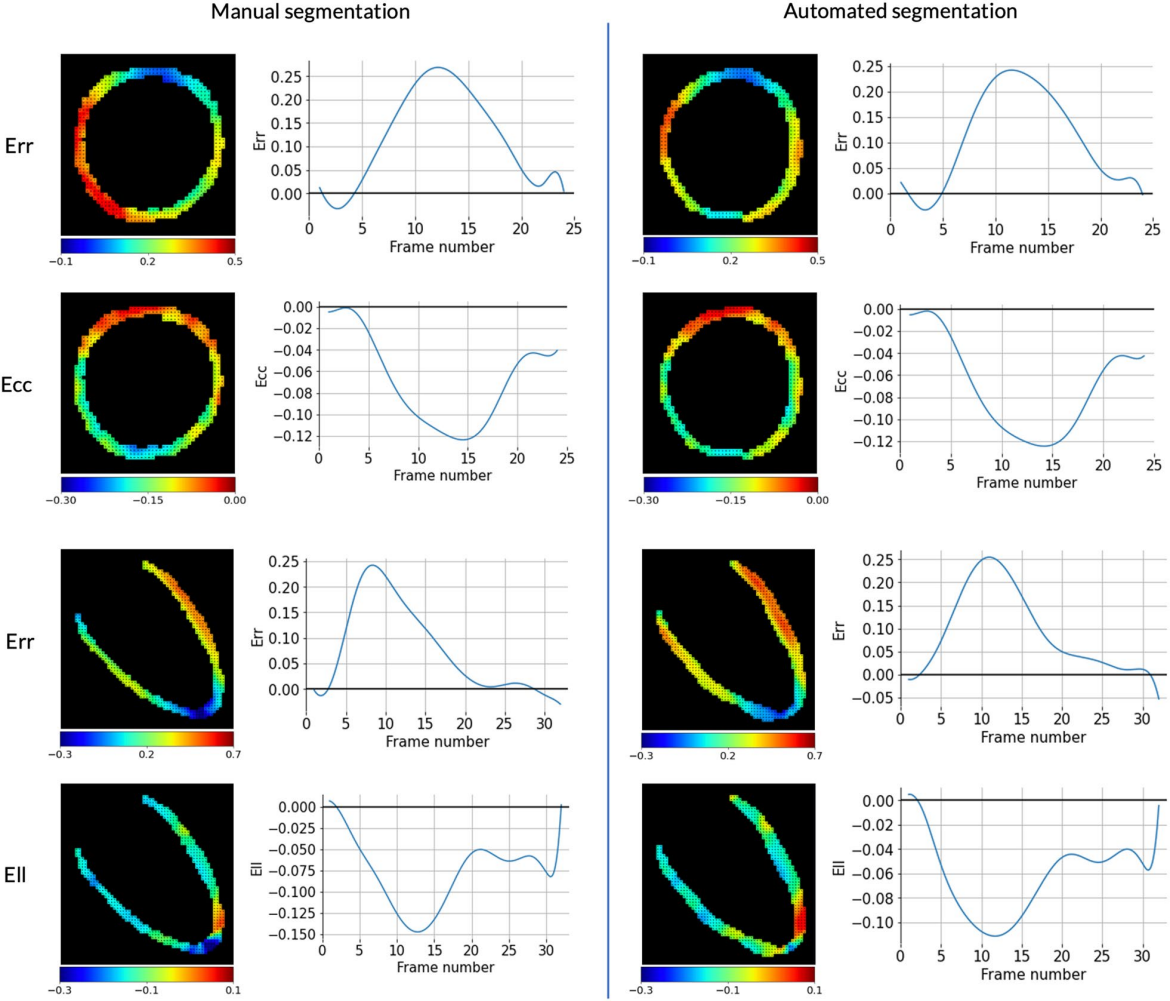
Typical strain results. Example of strain results obtained from manual or automated segmentation. Strain maps are displayed at peak strain, along with strain time curves

**Fig. 6:**
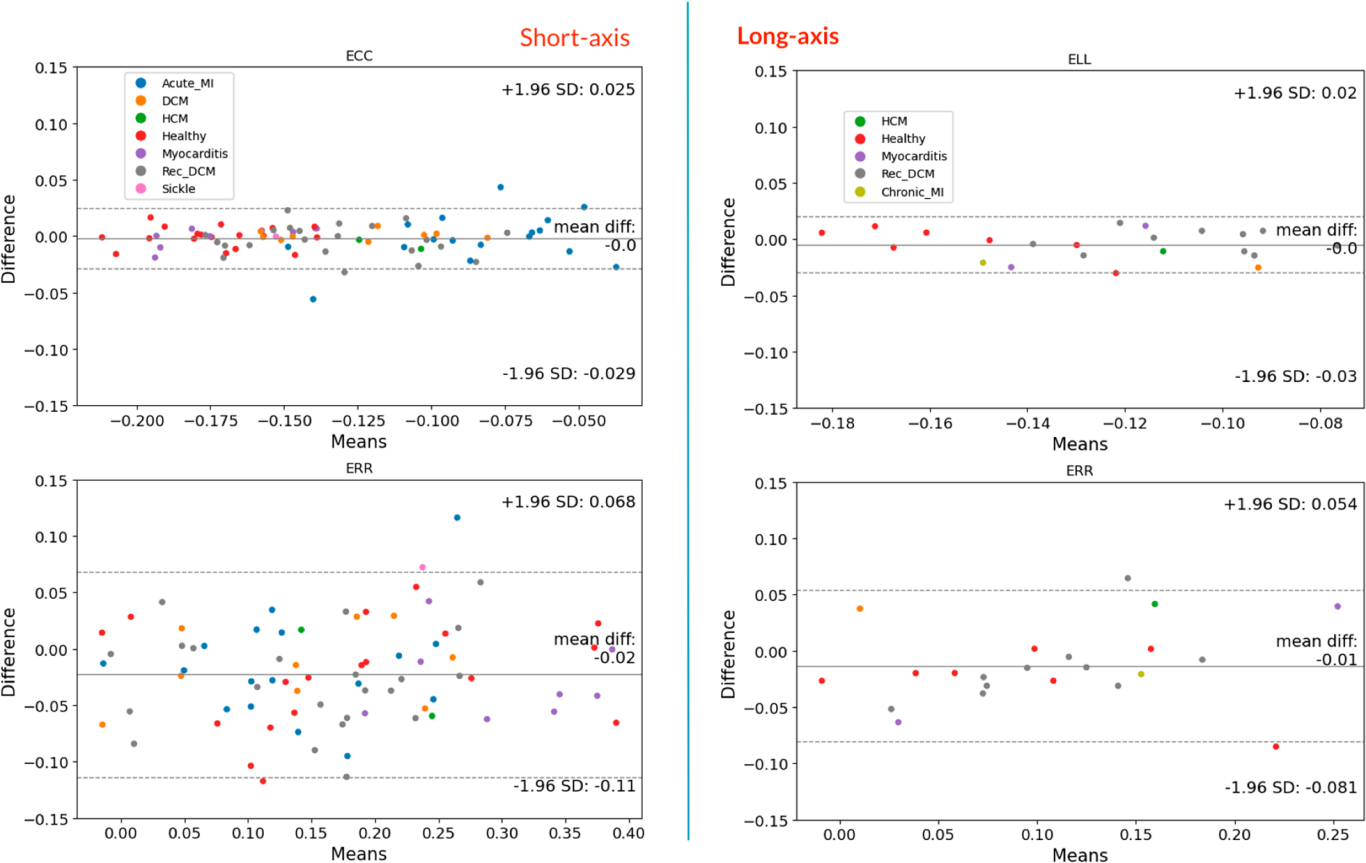
Bland–Altman plots, strain values after manual vs automated segmentation. Ecc, Ell and Err reproducibility when calculating strain with DENSEanalysis from manual vs automated LV myocardial segmentation. Left: short-axis. Right: long-axis. *Ecc* circumferential strain, *Ell* longitudinal strain, *Err* radial strain

After using the 2D + time DynU-Net architecture for extracting LV myocardium segmentations, inference results were processed using the described semi-automatic version of DENSEanalysis to extract Lagrangian strain and displacement values. Figure 5 shows typical examples of strain maps obtained either from manual or automated myocardial segmentation. To analyze these results, we compared peak strains calculated from strain time curves averaged over the whole LV myocardium in a slice to equivalent values obtained from a conventional pipeline with manual segmentation. Bland–Altman plots drawn from the test set results can be seen in Fig. 6. The bias was 0.00 for the SAx Ecc, with limits of agreement (± 1.96 SD) of -0.029 to 0.025, and respectively 0.02 and -0.11 to 0.068 for Err. The bias was 0.00 for the LAx Ell, with limits of agreement of -0.03 to 0.02, and respectively 0.01 and -0.081 to 0.054 for Err. We also computed CoV and ICC values to assess reproducibility between manual and automated analysis. As can be seen in Table 6, ICCs were considered excellent for all four strain analyses (0.95, 0.90, 0.91, 0.87 respectively for SAx Ecc, SAx Err, Ell, LAx Err). The CoV showed slightly different results, with an excellent CoV for Ecc and Ell (resp. 7.2% and 7.7%), while being considered fair for Err (21.2% and 24.6% for SAx and LAx datasets respectively).

**Table 6:** Agreement measures (Bland–Altman, CoV, ICC) of strain calculation between manually and automated left-ventricular myocardium segmentation

		Bias	Limits	CoV	ICC	ICC 95% CI
SAx	Ecc	0.00	− 0.03:0.03	7.2	0.95	0.92–0.97
	Err	0.02	− 0.11:0.07	21.2	0.90	0.85–0.94
LAx	Ell	0.00	− 0.03:0.02	7.7	0.91	0.79-0.96
	Err	0.01	− 0.08:0.05	24.6	0.87	0.72–0.95

*CoV* Coefficient of variation, *ICC* Intraclass correlation coefficient, *LAx* long axis, *SAx* short axis

Additionally, a transmural gradient in peak Ecc measured from short-axis views was reproduced by the DynU-Net model (p $=0.054$): the average subendocardial Ecc was $-0.157\pm 0.040$ and $-0.157\pm 0.042$ respectively for the automated and the ground-truth segmentation (p $=0.763$), and the average subepicardial Ecc was $-0.123\pm 0.035$ and $-0.125\pm 0.035$ (p $=0.030$).

### Generalization and reproducibility analysis

Our pipeline was tested on the second SAx independent test set, which was acquired in a different center (described in the methods). The 2D + time DynU-Net model achieved a Dice score of 0.84 ± 0.04 a Hausdorff distance of 4.2 ± 1.3 mm, averaged over all 45 SAx slices (from both 3T and 1.5T scanners).

**Fig. 7:**
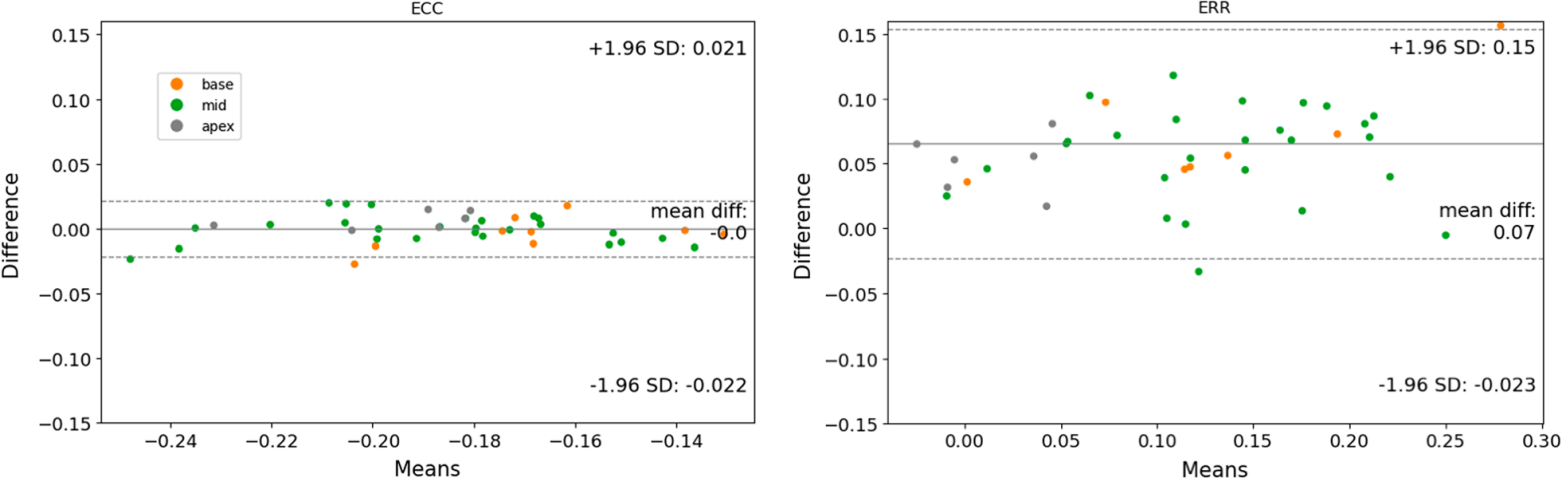
Bland–Altman plots, strain values after manual vs automated segmentation, short-axis external test set. Ecc and Err reproducibility when calculating strain with DENSEanalysis from manual vs automated LV myocardial segmentation. Ecc, circumferential strain; Err, radial strain; LV, left ventricular

After segmentation, images were processed in the same way as the original test set, to produce strain results and provide an analysis of the agreement between manual and automated segmentation. Bland–Altman plots can be see on Fig. 7. Bias and limits of agreement were 0.0 and − 0.022 to 0.021 for Ecc, respectively 0.07 and -0.023 to 0.15 for Err. As shown in Table 7, ICC was considered excellent for Ecc (0.92) and Err (0.85), while CoV was considered excellent for Ecc (4.2%) and poor for Err (47.0%).

**Table 7:** Agreement measures (Bland–Altman, CoV, ICC) of strain calculation between manually and automated left-ventricular myocardium segmentation, short-axis external test dataset

	Bias	Limits	CoV	ICC	ICC 95% CI
Ecc	0.00	− 0.02:0.02	4.2	0.92	0.86–0.96
Err	0.07	− 0.02:0.15	47.0	0.85	0.74–0.92

*CoV* Coefficient of variation *ICC* Intraclass correlation coefficient, *Ecc* circumferential strain, *Err* radial strain, *LV* left ventricular

**Fig. 8:**
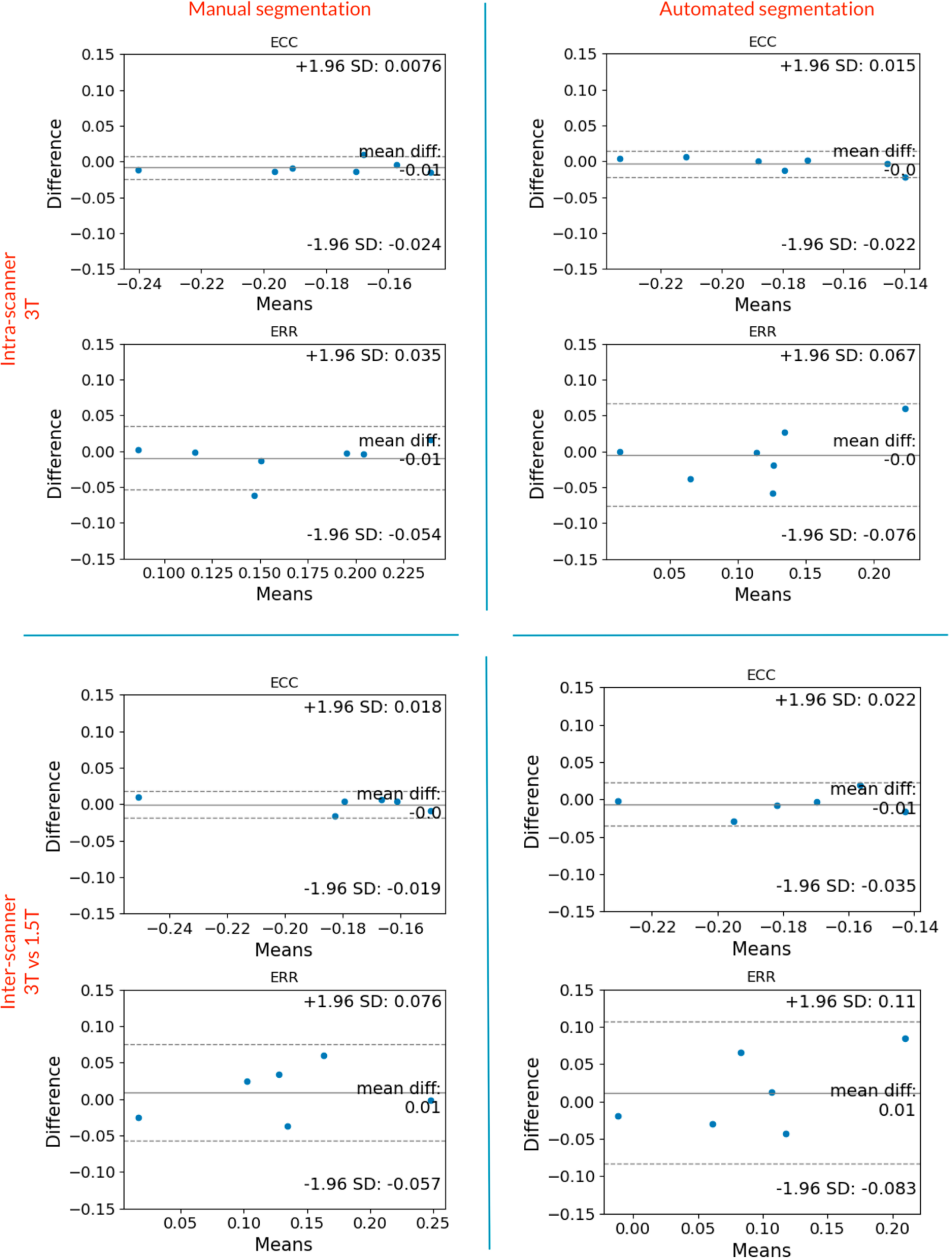
Bland–Altman plots, intra- and inter-scanner strain reproducibility. Analysis of the intra- and inter-scanner reproducibility in strain values, induced by manual or automated segmentation. Err and Ecc are reported for short-axis data. *Ecc* circumferential strain, *Err* radial strain, *LV* left ventricular

With this second dataset, we also analyzed the effect of the automated segmentation on the intra-scanner variability and inter-scanner variability, as mid SAx slices were acquired from multiple subjects on both a 1.5T and a 3T CMR scanner, with repeated scans. Reproducibility analysis of strain values can be found in Fig. 8.

## Discussion

We trained 2D and 2D + time nnU-Net models to segment the LV myocardium from DENSE magnitude images, training separate models for SAx and LAx datasets. 2D + time architectures were found to leverage temporal redundancies and remove segmentation artifacts over more simplistic 2D approaches. We re-implemented and simplified the nnU-Net framework using the MONAI library, based on the DynU-Net architecture. 2D + time DynU-Net models were successfully validated as they showed, when used in a complete pipeline to calculate Lagrangian strain, good agreement with manual analysis. The DENSEanalysis MATLAB tool was extended to automatically analyze full datasets with reduced processing time and minimal user interaction (from several minutes of manual processing, excluding segmentation, to less than 30 s). The modification of DENSEanalysis is a critical aspect of this study, as it can be used for further studies, even for standard manual processing. The generalizability of the models was validated by analyzing a second test set acquired from a different center and scan-rescan variability was quantified in multiple scanners.

In our work, we noticed that training 2D architectures based on individual frames only was sub-optimal. With cine DENSE, frames have decreasing contrast and brightness at later times in the cardiac cycle due to T1 recovery. While the inherent variability from this loss of contrast makes it harder for a network to perform well, its impact can be mitigated by implementing data augmentation with intensity-based transforms. However, the effects of other artifacts cannot be mitigated in this way. Early cine DENSE frames have poor contrast between blood and myocardium as the blood has not yet left the imaging slice and spiral streaking artifacts (sometimes rather substantial) may also be present in these frames. As a consequence, 2D networks frequently fail in the initial frames (see Fig. 3). Additionally, there is a significant reduction in myocardial signal over the cardiac cycle (p $<{10}^{-67}$ between mean LV myocardial signal in frame 1 vs mid-frame, and p $<{10}^{-113}$ for mid frame vs last frame), which further motivates the use of temporal networks.

Using 2D + time architectures enables the networks to leverage temporal redundancies in the image series. As we can see in Table 4, this first leads to a slight increase in average performance. More interestingly, we noticed that the cases noted above, where 2D segmentation fails on the early frames, are generally well segmented by the 2D + t architectures, as can be seen in Fig. 3. This trend over the cardiac cycle is shown in Fig. 2. While the blood-to-myocardium contrast is poor as expressed above, the 2D + time nnU-Net model is able to more accurately predict segmentation labels than its 2D counterpart.

This idea could be explored further. While we saw that temporal redundancies in a series of 2D images can be modeled by 3D convolutional architectures, these types of models might not be the best suited as the models are forced to learn temporal and spatial context using 3D convolutional kernels. Spatio-temporal relationships might be better modeled by other types of architectures, for example recurrent-based (RNN), by incorporating convolutional long short-term memory (LSTM) units in U-Net architectures as done by Lu et al*.* [31].

nnU-Net is an extensive framework that is currently the benchmark for numerous medical imaging tasks. It has been used in various challenges [13, 21–23, 58] was explored widely for cardiac segmentation [18, 19], and is frequently used as a base model in recent papers [6, 62]. However, nnU-Net has become highly complex with successive modifications, and shows limited flexibility for customization. Reproducing the entire nnU-Net pipeline in external software platforms, particularly on scanners where Python code is not normally supported, becomes challenging. Our MONAI implementation drastically simplifies the translation process, while showing results that are on par with nnU-Net. In addition, thanks to early stopping and caching options available in MONAI, we reduced the training time significantly.

We showed that the strain values calculated from DENSEanalysis were generally in excellent agreement when obtained from the manual ground-truth and the automated LV myocardium segmentation, particularly reproducing the Ecc transmural gradient in SAx views reported by Zhong et al*.* [64]. Additionally, when we compare to the results obtained from the recent multi-center reproducibility study [3], we can see that the strain agreements between the automated and manual segmentations are on par with the intra-user variability, which showed an excellent ICC across all strain components (0.93, 0.94, 0.92 resp. for Ecc, Ell, Err), and an excellent CoV for Ecc and Ell reproducibility but only fair for Err (resp. 3.0, 2.9 and 24.6). We can conclude, based on the strain comparability results on independent test sets, that the 2D + time DynU-Net inference does not induce a significant variability in the strain calculations. The quality of segmentation still plays an important part in the ability to calculate strain. For a limited number of cases, it happened that the contours produced at inference time by the trained 2D + time DynU-Net were not continuous, which made it impossible to analyze with DENSEanalysis to extract strain values (4 SAx and 3 LAx slices had this problem in the independent test set). This generally happens when the myocardium is particularly thin, especially in the LAx apical regions, or when the image contrast is reduced. From the perspective of doing inline quality control, when such a network will be used directly on the scanner, it is critical that the topology of the myocardial segmentation is preserved. To guide models in that respect, previous works have shown interesting results when topological priors are enforced during training time [7]. The impact of topological errors on the rest of the pipeline (phase-unwrapping and strain extraction) will however be mitigated when training additional models to automatically process these steps as well in future work.

We validated the trained DynU-Net architecture further on a second test set, from a different center, different scanners, and with different acquisition parameters. Despite these differences, the segmentation performance is on par with that found in the original test set, which is impressive given the domain shift. However, the most adverse cases, where Dice score and/or Hausdorff distance is impaired, show generally worse performance than those from the original test set, up to a point where segmentation can extend outside of the myocardium (Additional file 1: Fig. S2). More importantly, we see cases where the topology of the myocardium is lost, making the following strain analysis with DENSEanalysis impossible. Four of 45 SAx exams could not be processed because of these topological failures.

Additional work needs to be done to preserve the topological structure of the myocardial segmentation, as seen in the previous section, and this is particularly important to successfully conduct LAx analyses. We applied the long-axis 3D DynU-Net model on the second test set as well. While this resulted in reasonable segmentation performance (Dice score of 0.81 ± 0.04, Hausdorff distance of 14.5 ± 10.5), a majority of these cases showed a non-continuous topology, which made it impossible to conduct a strain analysis.

These segmentation issues mainly come from the distribution shift between the secondary test set and the original dataset. This shift is substantial, given that the data was acquired in a different center, on different scanners, and with different acquisition parameters. In particular, observers segmented a thicker myocardium as ground-truth compared to the original dataset, and the DENSE protocol typically acquired cardiac frames finishing just after peak systole, rather than filling the whole cardiac cycle. While the trained DynU-Net models are effective on the second test set, the distribution shift does impact performance, and domain adaptation strategies will prove necessary if we want to successfully export such networks to multiple centers. While intra- and inter-scanner variability are generally better with manual analysis, the variability introduced by the automated myocardial segmentation for short-axis data remains within reasonable limits. Indeed, the Bland–Altman reproducibility metrics (bias and limits of agreement) are either on par or superior to the ones exposed in the multi-center reproducibility study [3] for inter-observer variability. This is even more remarkable given the domain shift described above, which shows how promising 2D + time U-Net-based models can be for DENSE image analysis.

## Limitations

While the datasets used in this study are diverse, including subjects with various conditions, acquired and analyzed at different times, the quality of the ground-truth manual data was imperfect in many cases due to the limited amount of time available for the analyzing researcher to perform the manual analysis, as well as systematic differences between observers. In addition, the main dataset that we used to train our models was acquired from a single center on two different 3T scanners only. While we showed that, in terms of raw segmentation metrics, our models are able to mitigate the distribution shift that is introduced when using them for inference on external data, some topological problems remain and are necessary to be addressed if we want to successfully follow segmentation with strain extraction. Training on more diverse datasets, using domain adaptation solutions [14, 25, 39, 54] or enforcing topological coherence are potential strategies to mitigate this issue [7].

In this work, we explored how including temporal information in the DL models results in more robust segmentations of DENSE images than 2D models. However, DL was not applied to the rest of the pipeline, where minimal user interaction remains. In particular, current methods for phase unwrapping and strain estimation suffer from regularization and partial volume effects. In future work, we will extend the architectures developed in this study to understand how they might improve the processing of phase unwrapping and strain estimation, combined with the segmentation work presented in this study that will help future strain analysis. Ghadimi et al*.* [11] and Kar et al*.* [26] recently explored the use of 2D CNNs for automating the unwrapping of the myocardial phase, removing the need for seed points. It will be interesting to understand how temporal models can make robust end-to-end automated pipelines.

Finally, the development work that we have done on the models and DL pipeline was made with the aim of simplifying the portability of the process, especially for implementation on the scanner, for scan-time provision of strain maps. However, the actual impact on clinical pipelines is yet to be validated. In the future, we plan to test our models in inline settings and validate their use on clinical data.

## Conclusion

In this work, we trained DL networks to segment the LV myocardium from both SAx and LAx DENSE images for the first time, automating the most time-consuming step in the DENSE analysis. Temporal U-Net-based models show excellent robustness to contrast variability and acquisition artifacts when compared to manual methods, with no impairment of strain calculation if used in conventional DENSE analysis pipelines.

DL is extremely promising for the rapid and accurate extraction of cardiac strain information. Temporal models move the processing of DENSE sequences one step closer to the clinical setting, to estimate myocardial strain for patients suffering from cardiovascular diseases at the scanner, reducing the need for burdensome and time-consuming processing tasks, and improving the patient journey.

## Supplementary Information

**Additional file 1: Figure S1.** Quality Control application GUI. **Figure S2.** Failure modes from segmentation inference due to domain shift. **Figure S3.** Bland-Altman plots showing the variability introduced by the inference post-processing step. **Figure S4.** 3D DynU-Net architecture.

## Data Availability

The dataset used and analyzed during this study is available from the corresponding author on reasonable request.

## Abbreviations

CMR: Cardiovascular magnetic resonance
CoV: Coefficient of variation
CV: Cross-validation
DCM: Dilated cardiomyopathy
DENSE: Displacement encoding with stimulated echoes
DL: Deep learning
Ecc: Circumferential strain
ED: End-diastolic
Ell: Longitudinal strain
Err: Radial strain
ES: End-systolic
GUI: Graphical user interface
HCM: Hypertrophic cardiomyopathy
HD: Hausdorff distance
ICC: Intra-class correlation coefficient
LAx: Long-axis
LSTM: Long short-term memory
LV: Left ventricle/left ventricular
LVEF: Left ventricular ejection fraction
MCD: Myocarditis
MI: Myocardial infarction
RDCM: Recovered dilated cardiomyopathy
ReLU: Rectified linear activation function
RV: Right ventricle/right ventricular
SAx: Short-axis
SGD: Stochastic gradient descent optimizer
