# Supplementary material for: Automated segmentation of long and short axis DENSE cardiovascular magnetic resonance for myocardial strain analysis using spatio-temporal convolutional neural networks
Source: J Cardiovasc Magn Reson. 2023 Mar 30;25:16. doi: 10.1186/s12968-023-00927-y (PMC10061808; doi:10.1186/s12968-023-00927-y)
Supplement: Supplementary file 1 — Additional file 1: Figure S1. Quality Control application GUI. Figure S2. Failure modes from segmentation inference due to domain shift. Figure S3. Bland-Altman plots showing the variability introduced by the inference post-processing step. Figure S4. 3D DynU-Net architecture. [file 12968_2023_927_MOESM1_ESM.pdf]

Automated Segmentation of Long and Short Axis DENSE CMR for Myocardial Strain Analysis Using Spatio-temporal Convolutional Neural Networks

Additional file 1

1 Additional figures

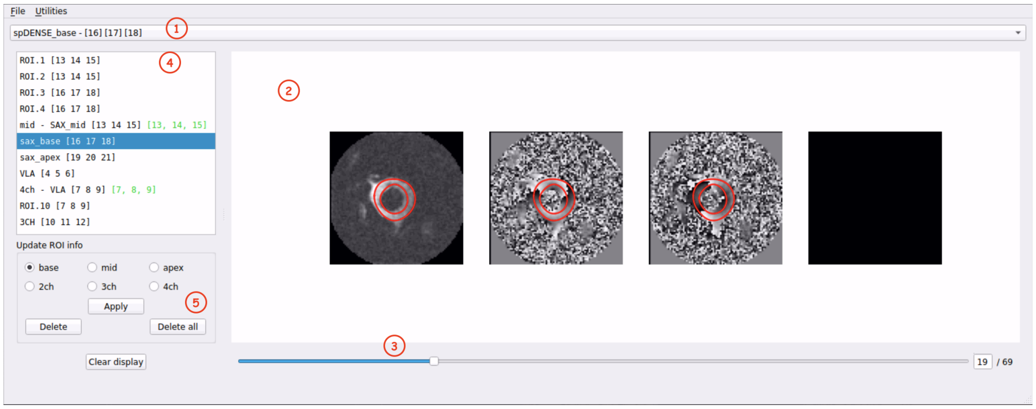

Figure S1 Quality Control application GUI. Subjects can be loaded and studied one by one. Drop-down menu (1) selects through the available scans for a given subject, showing sequence name and image indices. Panel (2) is the main image viewer, showing magnitude (first box) and phase images (two boxes for in-plane directions, last one for through-plane direction). Users can slide through each frame of the CINE sequence (3). Panel (4) shows manual labels for LV segmentation previously created with DENSEanalysis for the given subject (showing segmentation name and associated DENSE data slices). Selection will trigger the red myocardial borders displayed on images in panel (2). The main processing actions can be done from panel (5). By selecting a slice orientation and clicking apply, users have the possibility to change which DENSE images are associated with a selected region of interest, as well as indicating the correct view represented (base, mid, apex, two-chamber, three-chamber, four-chamber). This is delineated on panel (4) by the green indices and the view name prefix (example on row 5). By only applying these changes to good quality labels before saving, this process discards undesired manual segmentation and only keeps DENSE slices with appropriate labeling. Processing a single case (subject) generally takes from several seconds to a minute.

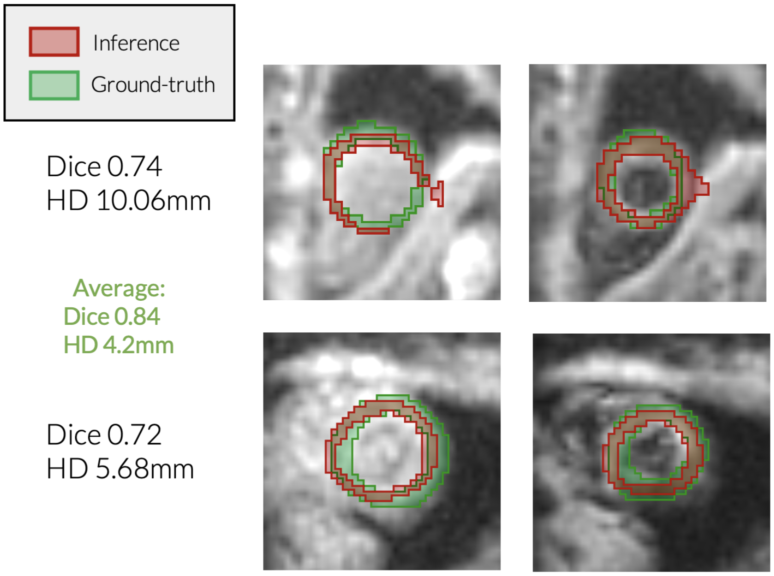

Figure S2 Failure modes from segmentation inference due to domain shift. Examples of short-axis segmentation results on cases from the second center. The top and bottom show inference examples with respectively impaired Hausdorff distance and impaired DICE score, at end-diastole (left column) and end-systole (right column).

2 Impact of converting segmentation maps into contours

As stated in the Methods, segmentation maps obtained from DynU-Net at inference time had to be post-processed in order to fit into DENSEanalysis. For that purpose, binary masks were transformed into myocardial contours. To demonstrate that this step has minimal impact on the rest of the pipeline, we transformed the ground-truth myocardial contours into binary masks and processed them back to retrieve myocardial contours. We then calculated strain values with DENSEanalysis from both the original smooth floating-point myocardial contours and those artificially engineered by reproducing the inference post-processing step, and compared the resulting strain components. The results are summarized in Table S1 and Figure S3. As we can see, variability for all components is within the limits of what was found in the multi-center reproducibility study [1], most of the variability measures being significantly lower than the reported intra-user variability.

Table S1 Agreement measures (Bland-Altman, CoV, ICC) showing the variability introduced by the inference post-processing step.

		Bias	Limits	CoV	ICC	ICC 95% CI
SA	Ecc	0.00	-0.03:0.02	7.2	0.95	0.92-0.97
	Err	0.01	-0.04:0.03	9.3	0.99	0.98-0.99
LA	Ell	0.00	-0.01:0.00	2.2	0.99	0.99-1.0
	Err	0.01	-0.03:0.01	10.2	0.99	0.97-0.99

CoV: Coefficient of variation, ICC: Intraclass correlation coefficient, CI: Confidence interval.

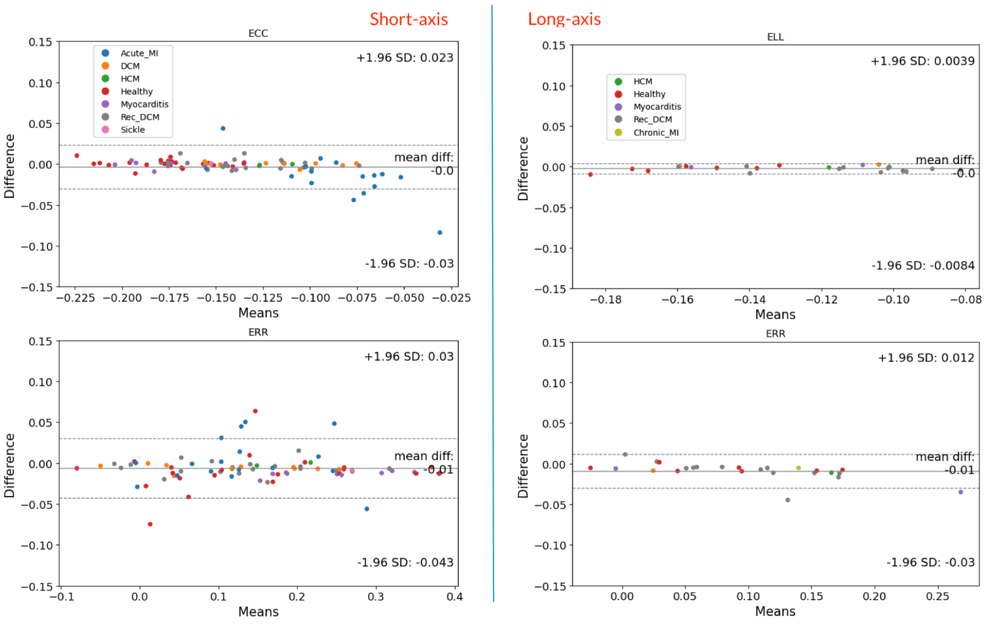

Figure S3 Bland-Altman plots showing the variability introduced by the inference post-processing step. Ecc, Ell and Err reproducibility when calculating strain with DENSEanalysis from manual floating-point contours either untouched or processed as a binary mask to reproduce the post-processing myocardium contouring step after deep learning inference. Left: short-axis. Right: long-axis.

3 Deep learning architecture

The 3D DynU-Net network (see Fig. S4) is composed of cascading layers in two successive paths: a contracting path for encoding context and a decoding path for localization. Each encoding layer is made of two sets of convolutional layers with kernel $3 \times 3 \times 3$, instance normalization [2] and a leaky rectified linear (ReLU) activation function. The first one of the two convolutions has a stride of 2, acting like a combined max pooling operation and reducing the size of the outputs by a factor 2. The number of feature maps in the first layer is 32, and increases by a factor 2 at each layer to get a bottleneck of size 1024 and 512, respectively for short-axis and long-axis datasets. For each decoding layer, a transpose convolution with kernel size $2 \times 2 \times 2$ and stride 2 is used to upsample the features maps. This is followed by two sets of convolutional layers with kernel $3 \times 3 \times 3$, instance normalization and a ReLU. To the input of each decoding layer is concatenated the output of the corresponding encoding layer, acting as skip-connections. To add to that U-Net-like architecture, deep supervision was implemented to further improve performance. The idea of deep supervision was first explored by Lee *et al.* in 2015 [3], and developed further in the case of U-Nets by Zhu *et al.* [4]. This forces the features learned by each hidden layer to be more semantically meaningful. In practice, deep supervision is implemented by adding a few convolutions at each decoding layer to produce segmentation estimates at different resolutions.

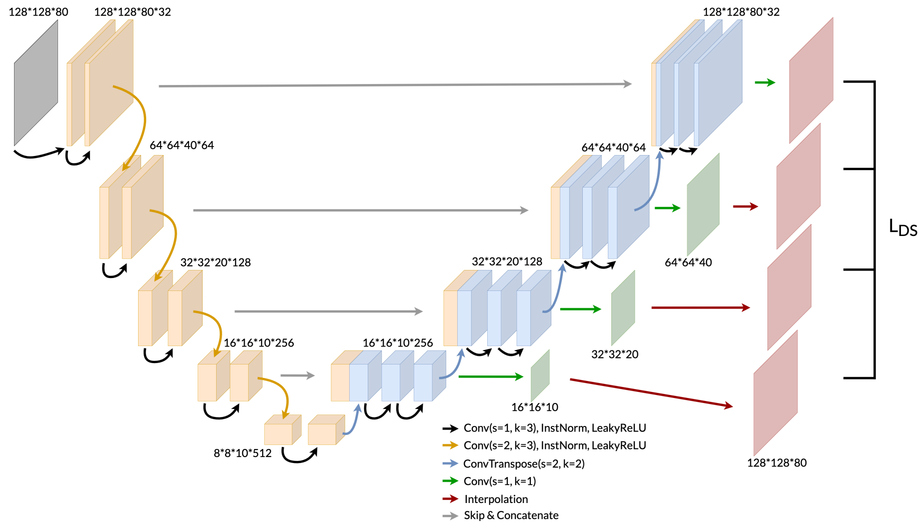

Figure S4 3D DynU-Net architecture. Like U-Net, the DynU-Net architecture has a contextual encoding path and a localization decoding path. Each decoding layer produces a segmentation estimate which is interpolated to match the input volume size. The various estimates are combined in a deep supervision loss (Eq. 4).

The network loss is a composition of Dice and Cross-Entropy losses, and is the weighted sum of sub-losses acting on the output of each decoding layer. The sub-losses can be expressed with:

$$L_{CE}(\mathbf{\hat{y}^n}, \mathbf{y}) = -\frac{1}{M}\sum_{i=1}^{M}[y_i \log(\hat{y}_i^n) + (1 - y_i)\log(\hat{y}_i^n)] \quad (1)$$

$$L_{DC}(\mathbf{\hat{y}^n}, \mathbf{y}) = 1 - 2\frac{\sum_{i=1}^{M} \hat{y}_i^n y_i}{\sum_{i=1}^{M} \hat{y}_i^n + y_i} \quad (2)$$

$$L_{DC-CE}(\mathbf{\hat{y}^n}, \mathbf{y}) = L_{DC}(\mathbf{\hat{y}^n}, \mathbf{y}) + L_{CE}(\mathbf{\hat{y}^n}, \mathbf{y}), \quad (3)$$

where L_{DC} is the Dice loss, L_{CE} the binary cross-entropy loss, $\mathbf{\hat{y}^n}$ the output probability estimate at layer n, $\mathbf{y}$ the ground-truth segmentation, $\mathbf{x_i}$ voxel value number i in image $\mathbf{x}$ (probability for output estimates or binary label for ground-truth segmentation), and M the number of voxels in the images. The general deep supervision network loss then becomes:

$$\mathcal{L}(\mathbf{\hat{y}^1}, \dots, \mathbf{\hat{y}^N}, \mathbf{y}) = \frac{1}{S}\sum_{n=1}^{N}\frac{1}{2^{(n-1)}} L_{DC-CE}(\mathbf{\hat{y}^n}, \mathbf{y}) \qquad S = \sum_{n=1}^{N}\frac{1}{2^{(n-1)}} \quad (4)$$

Data augmentation was performed by randomly applying transformations at each training epoch. Translations, rotations, Gaussian noise, Gaussian blur, intensity scaling and mirroring in any of the spatial dimensions were used. Images were padded to $128 \times 128 \times 80$ ($x \times y \times t$), and a 4-fold cross-validation was used as an ensemble strategy (and internally to optimize the training

hyperparameters). Like in nnU-Net, Stochastic Gradient Descent optimizer (SGD) with momentum was used for training, with an initial learning rate of 0.01, and a polynomial scheduler rate of $(1 - \text{super\_epoch}/\text{nbr\_super\_epoch})^{0.9}$ [5]. In nnU-Net, images are patched and each epoch corresponds to 250 iterations with patch batches; we define a "super-epoch" to be 7 epochs, so that the number of voxels processed by the network in a super epoch is in the same order of magnitude than the number of voxels processed by nnU-Net in an epoch. Every 7 epochs, validation performance is calculated, and the polynomial scheduler decreases the learning rate. The total number of super-epochs was 500. To reduce overfitting and speed up the training process, an early-stopping strategy was used based on the average dice score calculated for the validation set, with a patience of 50 super-epochs (training is stopped after 50 epochs if the validation performance does not improve). Network training was performed on an NVIDIA GeForce RTX 3090 GPU with 24 GB RAM, with a training time of around 27h for short-axis and 12h for long-axis.

To improve the accuracy of the segmentations during inference, a test-time augmentation strategy was used. 4 probability map estimates are generated by flipping the input images alongside every combination of the spatial axes, and the corresponding output maps are flipped back into the original reference space. The probability estimates are then averaged and thresholded to 0.5 to produce the final segmentation results.

References

1. Auger DA, Ghadimi S, Cai X, Reagan CE, Sun C, Abdi M, et al. Reproducibility of global and segmental myocardial strain using cine DENSE at 3 T: a multicenter cardiovascular magnetic resonance study in healthy subjects and patients with heart disease. Journal of Cardiovascular Magnetic Resonance. 2022;24(1):23.
2. Ulyanov D, Vedaldi A, Lempitsky VS. Instance Normalization: The Missing Ingredient for Fast Stylization; 2016. Available from: http://arxiv.org/abs/1607.08022.
3. Lee CY, Xie S, Gallagher PW, Zhang Z, Tu Z. Deeply-Supervised Nets. In: Proceedings of the Eighteenth International Conference on Artificial Intelligence and Statistics; 2015. p. 562–570.
4. Zhu Q, Du B, Turkbey B, Choyke PL, Yan P. Deeply-supervised CNN for prostate segmentation. In: Proceedings of the International Joint Conference on Neural Networks; 2017. p. 178–184.
5. Chen LC, Papandreou G, Kokkinos I, Murphy K, Yuille AL. DeepLab: Semantic Image Segmentation with Deep Convolutional Nets, Atrous Convolution, and Fully Connected CRFs. IEEE Transactions on Pattern Analysis and Machine Intelligence. 2018 4;40(4):834–848.
